# Metagenomes of Soils Samples from an Established Perennial Cropping System of Asparagus Treated with Biostimulants in Southern France

**DOI:** 10.1128/genomeA.00511-17

**Published:** 2017-06-15

**Authors:** Julien Crovadore, Ali Asaff Torres, Raúl Rodríguez Heredia, Bastien Cochard, Romain Chablais, François Lefort

**Affiliations:** a Plants and Pathogens Group, Institute Land Nature and Environment, hepia, HES-SO University of Applied Sciences and Arts Western Switzerland, Jussy, Geneva, Switzerland; b Centro de Investigación en Alimentación y Desarrollo, A.C., Hermosillo, Sonora, Mexico; c Innovak Global, Productos Quimicos de Chihuahua S.A. de S.V., Chihuahua, Chihuahua, Mexico

## Abstract

We report here the metagenomes of soils samples from a perennial cropping system of asparagus that was treated with two biostimulants. Two treatments were compared to an untreated control. Control soils samples were taken at the beginning and at end of the experiment.

## GENOME ANNOUNCEMENT

Metagenomic studies of soils usually attempt to correlate the composition of the soil microbiota with their functions or their functional potential (1–5). These studies have even revealed different microbiota compositions in various agricultural systems, such as those using conventional tillage and no tillage (2, 3), cultivated and noncultivated soils (2, 4), rotated and nonrotated crops (3), organic and conventional agriculture (5), and treatment with fertilization (6). 

In the present study, a perennial cropping system of asparagus (Darlise variety) located at Aimargues, France, was established in sandy and silty soil, and the crops were treated with two biostimulants, ExuRoot (Innovak Global, Mexico) and Cérès (Biovitis, France), which were applied four times between mid-July and mid-September 2016. Sampling was carried out in June 2016, prior to the application of the biostimulants, and again in September, after they were applied. For each of the three modalities (i.e., no treatment, ExuRoot, and ExuRoot + Cérès) and their repetitions, 50 g of rhizosphere soil were obtained with a sterile shovel from 10 sampling points at depths between 20 and 40 cm. Following the same methodology, the control modality was sampled twice: at the beginning of the experiment and at the end. The pooled samples were kept in plastic containers at −80°C until DNA extraction. After thawing and homogenization, subsamples (10 g) were disrupted with TissueLyser II (Qiagen, Germany). Metagenomic DNA samples were extracted using the PowerSoil DNA isolation kit (Mo Bio, Inc./Qiagen, USA). Quality and quantity controls were performed by gel electrophoresis, spectrophotometry (Nanodrop ND-1000), and fluorometry (Qubit version 3.0). One microgram of metagenomic DNA was sheared to an average fragment size of 350 bp in an AFA microtube (Covaris, USA) using an S2 ultrasonicator (Covaris). Libraries were produced with the TruSeq DNA PCR-free library kit (Illumina). Whole-metagenome shotgun sequencing was carried out within two high-output (300 cycles) Illumina MiniSeq runs using 2 × 150-bp paired-end reads. BaseSpace (Illumina) was used to extract the reads and to trim adaptors and Ns, and FastQC (http://www.bioinformatics.babraham.ac.uk/projects/fastqc) was used to perform the quality control. Sequencing yielded between 3,360,000 reads (0.5 Gb) and 12,350,000 reads (1.85 Gb) per sample. One Codex (7), Kaiju (8), and the MG-RAST (MetaGenomics Rapid Annotations using Subsystems Technology) pipeline (9) were used for the analysis of the bioinformatics and the identification of operational taxonomic units (OTUs). 

A wide diversity of OTUs was retrieved from these soil samples, in which the classes *Alphaproteobacteria* and *Actinobacteria* were dominant. The 10 most abundant genera, representing between 29.43% and 42.8% of all species in all samples, were *Bradyrhizobium*, *Nocardioides*, *Mycobacterium*, *Rhizobium*, *Streptomyces*, *Mesorhizobium*, *Microbacterium*, *Pseudomonas*, and *Sphingomonas*. Three of them (*Bradyrhizobium*, *Mesorhizobium*, and *Streptomyces*) had already been observed as prominent genera in metagenomic studies of soils of sugar beet cultures (10, 11). Most of the genera found here are known to host plant growth–promoting rhizobacteria species, such as those belonging to the genera *Bradyrhizobium*, *Rhizobium*, and *Mesorhizobium*, and showed important diversity, with dozens of different OTUs in each sample.

### Accession number(s).

The raw sequencing data of the metagenomes have been made publicly available through the NCBI’s Sequence Read Archive (SRA) (https://doi.org/10.1093/nar/gkq1019) under the SRA accession numbers given in Table 1. They have also been deposited in the MG-RAST database (http://metagenomics.anl.gov).

**TABLE 1:** Sequence Read Archive (SRA) accession numbers

Sample name	SRA accession no.
Soil metagenome of an asparagus culture, initial control R1	SRR5381855
Soil metagenome of an asparagus field culture, initial control R2	SRR5381878
Soil metagenome of an asparagus field culture, initial control R3	SRR5381880
Soil metagenome of an asparagus field culture final control R1	SRR5381886
Soil metagenome of an asparagus field culture final control R2	SRR5381887
Soil metagenome of an asparagus field culture, final control R3	SRR5381892
Soil metagenome of an asparagus field culture, treated with ExuRoot R1	SRR5381894
Soil metagenome of an asparagus field culture, treated with ExuRoot R2	SRR5381897
Soil metagenome of an asparagus field culture, treated with ExuRoot R3	SRR5381899
Soil metagenome of an asparagus field culture, treated with ExuRoot and Cérès R1	SRR5381902
Soil metagenome of an asparagus field culture, treated with ExuRoot and Cérès R2	SRR5381903
Soil metagenome of an asparagus field culture, treated with ExuRoot and Cérès R3	SRR5381907
